# MicroRNA-135b/CAMK2D Axis Contribute to Malignant Progression of Gastric Cancer through EMT Process Remodeling

**DOI:** 10.7150/ijbs.58062

**Published:** 2021-05-10

**Authors:** Longtao Huangfu, Qifei He, Jing Han, Jingyao Shi, Xiaomei Li, Xiaojing Cheng, Ting Guo, Hong Du, Wanhong Zhang, Xiangyu Gao, Fengming Luan, Xiaofang Xing, Jiafu Ji

**Affiliations:** 1 Key Laboratory of Carcinogenesis and Translational Research (Ministry of Education), Division of Gastrointestinal Cancer Translational Research Laboratory, Peking University Cancer Hospital, Fu-Cheng Road, Beijing, 100142, China.; 2 Department of Gastrointestinal Surgery, Peking University Cancer Hospital, Beijing, Fu-Cheng Road, Beijing, 100142, China.; 3 Center of Minimally Invasive Gastrointestinal Surgery, Shanxi Cancer Hospital, Zhigong New Street, Taiyuan, Shanxi, China.; 4 Department of Orthopedics, The First Affiliated Hospital of Shenzhen University, Shenzhen Second People's Hospital, Shenzhen, 518000, China.

**Keywords:** MicroRNA-135b, CAMK2D, EMT, Oligonucleotide therapy, Gastric cancer.

## Abstract

There is a continued need for investigating the roles of microRNAs (miRNAs) and their targets on the progression of gastric cancer (GC), especially metastasis. Here, we performed an integrated study to identify dysregulated miRNAs critical for GC development and progression. miR-135b was determined as a promising biomarker for GC. The expression level of miR-135b was increased among GC cell lines, patient tumor tissues, serum samples, and correlation with aggravation of the GC patients. The *in vitro* functional assays demonstrated overexpression of miR-135b promoted cell proliferation, migration and invasion in GC, while miR-135b inhibition led to the opposite results. CAMK2D was found to be the direct target of miR-135b, serving as a tumor suppressor in GC cells. Based on our and public datasets, we confirmed the attenuation of CAMK2D expression in GC tissues. And, the expression levels of miR-135b and CAMK2D were closely associated with prognosis of GC patients. Ectopic expression of miR-135b resulted in the down-regulation of CAMK2D. Additionally, CAMK2D was a prerequisite for miR-135b to promote GC cells proliferation and migration by regulating the EMT process, which was confirmed by the *in vivo* experiments. Importantly, *in vivo* injection of miR-135b antagomir significantly repressed the tumor growth and metastasis of xenograft models, which suggested that the miR-135b antagomir were promising for clinical applications. Taken together, these results indicate that miR-135b/CAMK2D axis drives GC progression by EMT process remodeling, suggesting that miR-135b may be utilized as a new therapeutic target and prognostic marker for GC patients.

## Introduction

Gastric cancer (GC) is the sixth common cause of cancer globally, with 1,090,000 new cases each year, accounting for 5.6% of all cancers, and the third lead cause of cancer-related human mortalities 1. More than 70% of patients with early GC have no symptoms or only mild symptoms, and patients with advanced GC may experience abdominal discomfort or pain 2. Regardless of the intensive effort that has been spent on developing early detection methods and novel therapeutic targets, the overall 5-year survival remained low 3, 4. The efficacy of conventional surgery and radiotherapy for progressive GC is not satisfactory, and malignant growth and systemic metastasis are the major reasons for the unsatisfactory survival of GC patients in the advanced stage 5. Therefore, it will be critical for GC treatment that clarifying the molecular mechanisms for the development and progression of GC and identifying novel therapeutic targets.

MicroRNAs (miRNAs) are small non-coding RNA molecules (about 22 nt long) that that can act as post-transcriptional regulators of gene expression 6. Dysfunction of miRNA expression was observed in multiple human diseases, especially malignant tumors. Many of them are annotated as “oncomirs” or tumor “suppressors” since regulating the expression of cancer-related genes at the post-transcription level 7. Especially, many miRNAs had been found to participate in the progression of GC, such as miR-1, miR-7, miR-23a, and miR-520f 8. More and more miRNAs have been suggested to be closely associated with migration and invasion of GC cells, with the abnormal expressions in cancer tissues and cell lines. For instance, it is reported that decreased miR-7 enhances GC metastasis by up-regulating expression of IGF1R and NF-κB RelA/p65 9, 10. miR-216a inhibits the metastasis of GC by targeting JAK2/STAT3-mediated EMT process. miR-1258 acts as a tumor suppressor to inhibit GC progression by targeting HPSE, and may serve as a novel biomarker and therapeutic target in the treatment of GC 11. Therefore, ongoing research in this area is worthwhile.

Recently, microRNA-135b (miR-135b) has been repeatedly reported in cancer-related studies, as an oncogene, found increased in several malignant tumors and promotes cancer cell proliferation by targeting tumor suppressors 12-14. For example, miR-135b was reported to promote tumor progression by modulating the PTEN/PI3K pathway or TGF-β pathway in colorectal cancer 15, 16. MiR-135b was also demonstrated mediates gemcitabine sensitivity in breast cancer cells by modulating mTOR signaling pathway 17. Upon these researches, highly expressed miR-135b involves in promotion or repression of tumorigenesis and tumor progression by modulating tumor cells proliferation, invasion and migration and so on. Although miR-135b has been reported to be dysregulated in GC, the molecular mechanism of miR-135b in cancer metastasis was not fully uncovered.

Calcium/calmodulin-dependent protein kinase type II delta (CAMK2D) is a tumor suppressor that mediates the anti-proliferative and differentiating, at least in part through actions of calcium transduction 18, 19. Interestingly, recent studies revealed that members of CAMK2 family were implicated in the regulation of epithelial to mesenchymal transition (EMT) process through activation of MAPK and Notch-1 pathways 20, 21. Moreover, CAMK2D was served as a potential prognostic marker for overall survival of early-stage non-small cell lung cancer (NSCLC) in Chinese populations 22. It is well established that the expression levels of the CAMK2D are reduced, or even lost during tumorigenesis 23-25. However, the biological function and the epigenetic regulation of CAMK2D in gastric cancer are not fully understood.

In the present study, we performed an integrated analysis to identify GC-associated dysregulated miRNAs critical for GC development and progression. miR-135b was found to be upregulated among the GC cell lines, and the tumor tissues and blood samples derived from GC patients. Based on bioinformatics prediction, CAMK2D was found to be a potential binding target for miR-135b. Moreover, we found a significant negative correlation between the expression levels of miR-135b and CAMK2D. The current study was designed to investigate whether miR-135b promote GC metastasis with the aim of identifying new therapeutic strategies as well as the underlying molecular mechanisms associated with CAMK2D.

## Results

### The level of miR-135b was upregulated in GC patient samples

**Figure 1:**
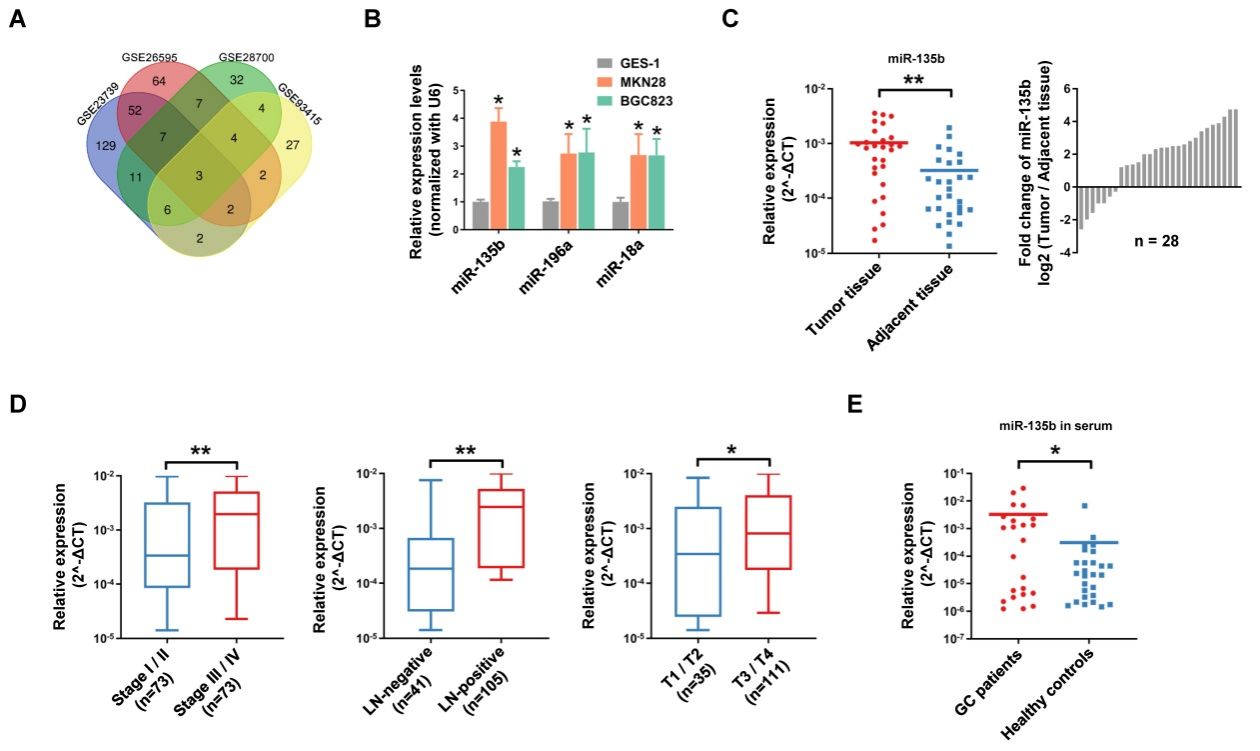
miR-135b was frequently upregulated in GCs and promoted GC cells malignancy. (A) Venn diagram of significant upregulated miRNAs in GC datasets (Log_2_[fold change]>2, *P*<0.05). miR-135b, miR-196a and miR-18a were overlapped among 4 GC datasets (Open source from https://www.ncbi.nlm.nih.gov/geo. The accession number: GSE23739, GSE26595, GSE28700, GSE93415). (B) The expression levels of miR-135b, miR-196a and miR-18a in MKN28, BGC823 and GES-1 cells were detected by qRT-PCR. n=6 independent experiments. *P<0.05 *vs* GES-1. (C) qRT-PCR analysis for miR-135b expression in 28 paired primary GC tissues and adjacent tissues (left panel). Ratio (tumor tissue/adjacent tissue) of miR-135b expression in 28 paired primary GC tissues and adjacent tissues (right panel). U6 was used as an internal control. (D) The relationship between miR-135b expression and clinical stages, lymph node-metastasis or local invasion. (E) The level of circulating miR-135b in the serum samples. The serum samples of GC patients were collected before operation (n=23) and the healthy controls taken from a physical examination (n=27). Let-7e was used as an internal control. *P<0.05, **P<0.01.

Firstly, we conducted a comprehensive comparative analysis of miRNA expression in tumor tissues and normal tissues in 4 independent GEO datasets (Open source from https://www.ncbi.nlm.nih.gov/geo. The accession number: GSE23739, GSE26595, GSE28700, GSE93415). After merging these data sets, 3 consistently upregulated miRNAs miR-135b (MIMAT0000758), miR-196a (MIMAT0000226) and miR-18a (MIMAT0000072) were identified (Figure 1A). Further study showed that miR-135b, miR-196a and miR-18a were significantly upregulated in GC cell lines MKN28 and BGC823 compared with that in the gastric mucosal epithelial cell line GES-1 (Figure 1B). Among these miRNAs, miR-135b seemed a promising diagnostic marker for a variety of cancers, but there was not such a finding in GC (Figure S1A and S1B). Next, we validated the expression level of miR-135b in 28 paired GC tissues and adjacent tissues. The results confirmed that miR-135b was markedly upregulated in GC tissues (Figure 1C). In order to elucidate whether miR-135b expression level was related to GC progression, we determine the association between miR-135b and clinicopathologic status in 146 cases of GC patients. The statistical analysis showed that a strong correlation between miR-135b level and TNM stage, lymph-node metastasis, and local invasion (Figure 1D). Interestingly, the level of circulating miR-135b in the serum sample was also significantly increased in GC patients compared to healthy controls (Figure 1E). Collectively, these findings suggested that a high level of miR-135b was positively associated with malignant progression and might be a promising diagnostic biomarker of GC.

### Overexpression of miR-135b enhanced the abilities of GC cells proliferation, migration and invasion

**Figure 2:**
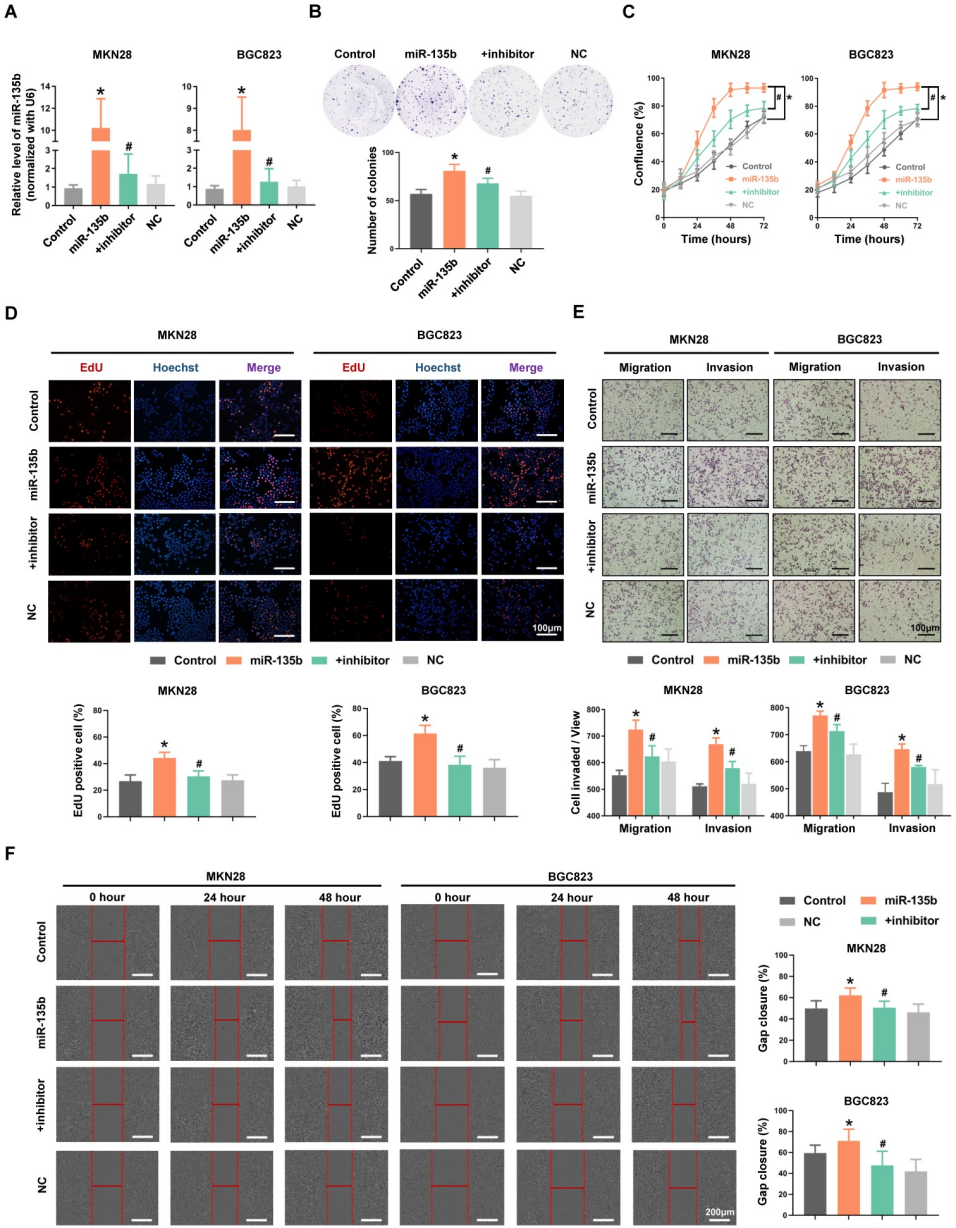
(A) MKN28 and BGC823 were transfected with miR-135b mimics, negative control (NC), or co-transfected with both miR-135b mimics and its inhibitor respectively. The transfection efficiency was further confirmed by qRT-PCR. (B) Cell proliferation was measured by IncuCyte live cell analysis system. (C) Colony formation assay. Overexpression of miR-135b increased the number of colony formation, while inhibition of miR-135b reversed this action. (D) The activity of DNA replication was detected by EdU staining. (E) MKN28 and BGC823 cells were determined for their migration capability by transwell assay. (F) Cell migration was tested using monolayer wound healing assay. Scale bar as indicated. n=6 independent experiments. *P<0.05 *vs* Control, #*P*<0.05 *vs* miR-135b.

To explore the potential role of miR-135b in GC pathogenesis, MKN28 and BGC823 cells were transfected with miR-135b mimics or co-transfected both miR-135b mimics and inhibitor. The efficiency of transfection was confirmed by qRT-PCR (Figure 2A). We found that overexpression of miR-135b enhanced the ability of colony formation in MKN28 cells, while inhibition of miR-135b in these overexpressed cells reversed this action (Figure 2B). In addition, upregulation of miR-135b promoted the proliferation of both MKN28 and BGC823 cells (Figure 2C). And, the activity of DNA replication in these cells was also increased upon miR-135b overexpression (Figure 2D). However, the reduction of miR-135b expression inhibited GC cells proliferation and decreased the activity of DNA replication. Next, we conducted transwell and wound healing assays to determine the involvement of miR-135b in cell migration and invasion, which represented that the increased level of miR-135b enhanced the abilities of both migration and invasion in these cells. Simultaneously, reversed upon the expression of miR-135b could restore both migration and invasion in GC cells (Figure 2E and 2F). These results demonstrated that miR-135b served as an oncogene in GC, and was involved in the regulation of cell proliferation and migration.

### CAMK2D was a direct target of miR-135b

**Figure 3:**
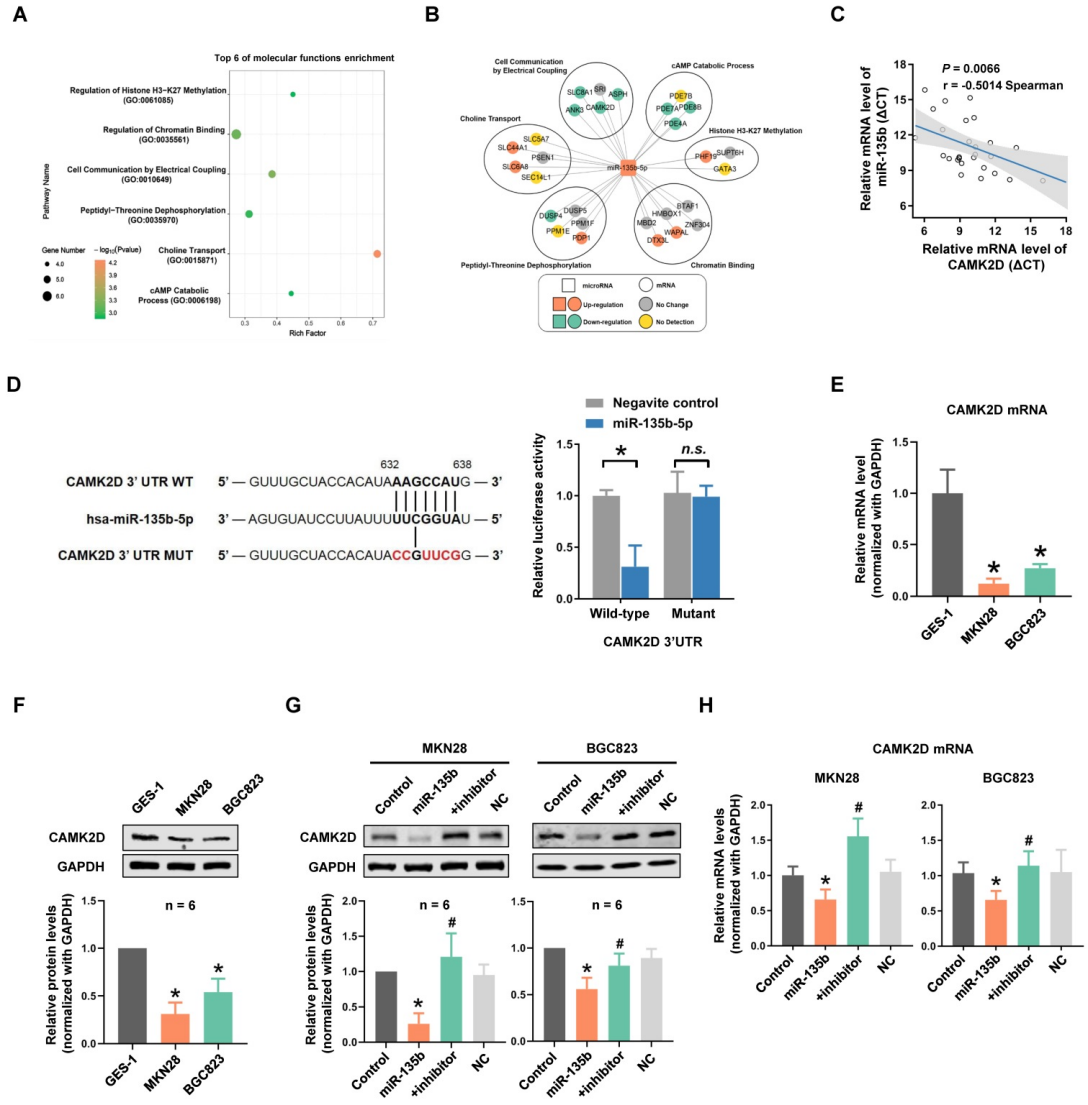
CAMK2D was the direct target of miR-135b. (A) Molecular functions enrichment analysis of the targets of miR-135b. Only the top six enriched GO terms are represented, GO terms with their *P* value, rich factor, and gene count. (B) The network of relationship among miR-135b and its targets. Most of the target genes in the term of cell communication were upregulated. (C) The negative correlation between CAMK2D and miR-135b expression levels in GC tissues (n=28). (D) A putative miR-135b binding site in the 3'UTR of CAMK2D is indicated in bold (left panel). HEK293T cells were cotransfected with NC or miR-135b mimics, and luciferase reporter plasmid carrying either wild-type or mutant 3'UTR of CAMK2D (right panel). (E) The mRNA level of CAMK2D in MKN28, BGC823 and GES-1 cells. (F) The protein expression level of CAMK2D in MKN28, BGC823 and GES-1 cells. (G) GC cells were transfected with miR-135b mimics alone or both mimics and inhibitor. After 48 hours, the CAMK2D protein expression was further detected by western blot. GAPDH was used as an internal control. (H) The mRNA level of CAMK2D after 36 hours GC cells transfected. n=6 independent experiments. *P<0.05 *vs* GES-1 or Control, #*P*<0.05 *vs* miR-135b.

To clarify the potential role and mechanism of miR-135b in GC progression and metastasis, the target genes of miR-135b were predicted by TargetScan and were listed to gene ontology (GO) annotation dataset for analysis of molecular functions. GO enrichment analysis showed that target genes of miR-135b were enriched in the molecular functions of histone H3-K27 methylation, chromatin binding, cell communication by electrical coupling (Figure 3A). We next analyzed the expression levels of predicted target genes using Peking University Cancer Hospital Gastric Cancer Transcriptome Dataset (PUCH dataset). Interestingly, most of the target genes in the term of cell communication were upregulated (Figure 3B). Among these genes, CAMK2D was considered to be a tumor suppressor, involved in the proliferation and migration of tumor cells. The correlation expression between miR-135b and CAMK2D in GC tissues were further determined by qRT-PCR and we found a significant negative correlation between them (r = -0.50, *P* = 0.0066; Figure 3C, Figure S2). Therefore, the counteraction of miR-135b and CAMK2D in regulating the cell malignant phenotypes derived us to study the miRNA-target relationship between miR-135b and CAMK2D.

To further investigate whether CAMK2D was directly targeted by miR-135b, a luciferase reporter system was constructed, which contained either the wild-type (WT) or mutant (MUT) binding site of miR-135b in the CAMK2D 3'UTR. Our results showed that the cells cotransfected with both miR-135b mimics and CAMK2D-WT reporter exhibited a significant reduction in luciferase activities. On the contrary, no significant change was observed in the luciferase activities of CAMK2D-MUT reporter between miR-135b and negative control groups (Figure 3D). We next tested whether overexpression of miR-135b affected CAMK2D expression. Both the mRNA and the protein levels of CAMK2D in GC cells were markedly reduced compared with GES-1 cells (Figure 3E and 3F). Of note, the decrease in both protein and mRNA levels of CAMK2D upon miR-135b overexpression, and a marked increase in CAMK2D level following miR-135b inhibition were found in GC cells (Figure 3G and 3H). Taken together, these results indicated that CAMK2D was a direct target of miR-135b in GC cells and could had an important function for GC progress.

### CAMK2D was downregulated in GC tissues and was significantly associated with poor prognosis

**Figure 4:**
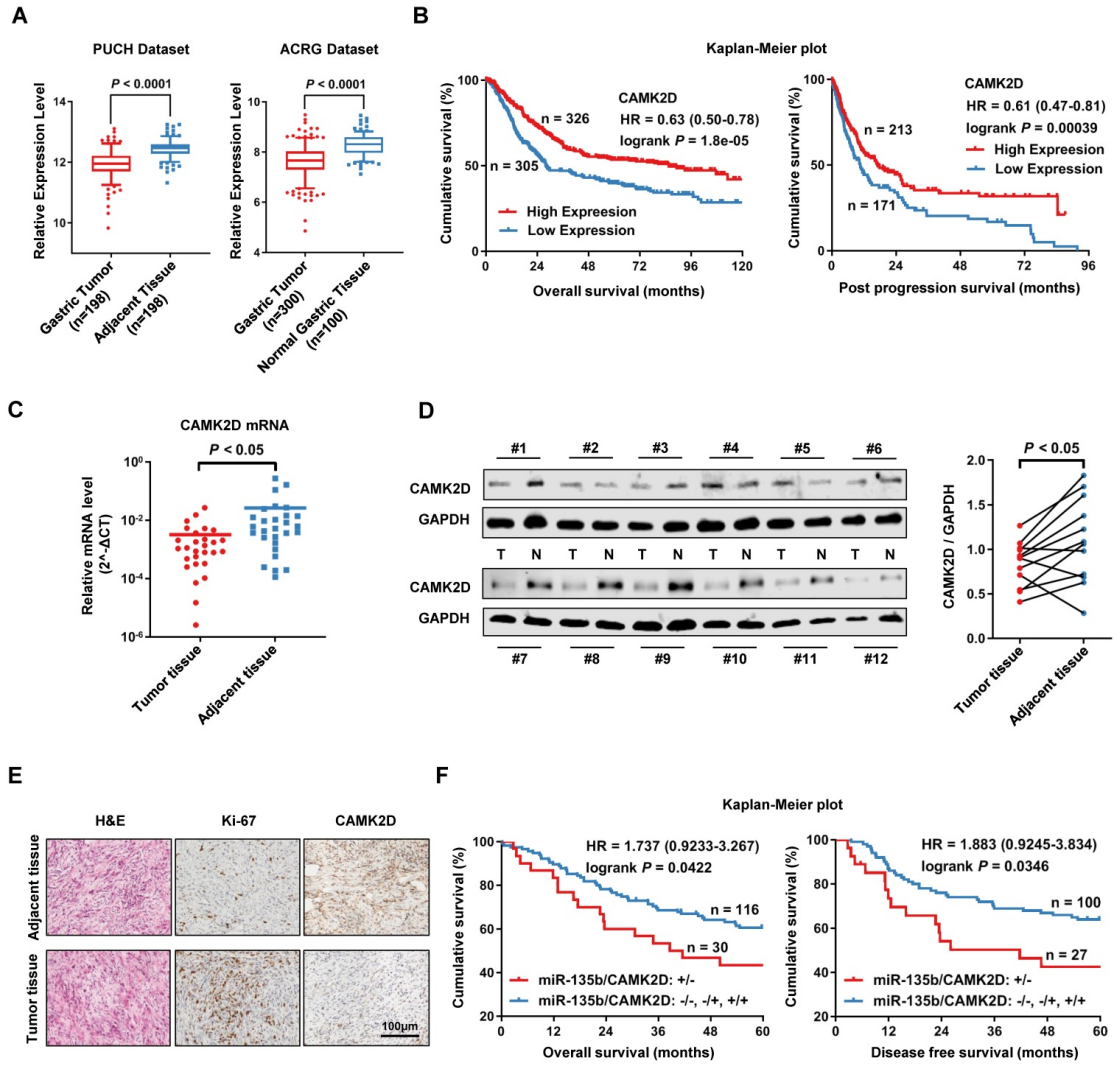
CAMK2D was downregulated in GC tissues and was associated with poor prognosis of GC patients. (A) The expression level of CAMK2D was markedly increased in GC tissues compared with adjacent tissues or normal gastric mucosa in Peking University Cancer Hospital Gastric Cancer Transcriptome Dataset (PUCH dataset) and ACRG dataset (GSE66229). (B) Kaplan-Meier survival analysis of overall survival (left panel) and post progression survival (right panel) obtained from public gene expression datasets. (C) The mRNA level of CAMK2D in 28 paired primary GC tissues and adjacent tissues was detected by qRT-PCR. (D) Western blot analysis for CAMK2D expression in 12 paired GC tissues and adjacent tissues. Student's *t* tests were performed. (E) H&E staining and IHC staining of Ki-67 or CAMK2D in GC tissues from patients. (F) Survival analysis of overall survival (left panel) and disease-free survival (right panel) in 146 cases of GC patients. The median expression value was used as the cutoff. HR: Hazard ratio.

Using PUCH dataset and ACRG dataset, we further confirmed that the expression of CAMK2D was significantly lower in GC tissues than in normal tissues (Figure 4A). We also found that the low expression level of CAMK2D was significantly associated with poor prognosis in patients with GC, as assessed using KM plotter online 26 (Figure 4B). Next, we measure CAMK2D expression in GC tissues and adjacent tissues. Indeed, the mRNA level of CAMK2D was found to be significantly lower in GC tissues than adjacent normal tissues (Figure 4C). Additionally, the protein level of CAMK2D was mainly decreased in tumor tissues (Figure 4D), as well as confirmed by immunohistochemistry (IHC) staining (Figure 4E). In our cohort study, GC patients exhibiting upregulation of miR-135 with downregulation of CAMK2D showed a strong association with decreased 5-year OS and DFS (Figure 4F). However, the expression level of miR-135b or CAMK2D alone was only weakly correlated with the prognosis in GC patients (Figure S3). These data suggested that CAMK2D was a potential prognostic marker of gastric cancer and may have important functions during tumor progression and metastasis.

### CAMK2D-mediated EMT process was essential for the miR-135b-induced GC cells malignancy

**Figure 5:**
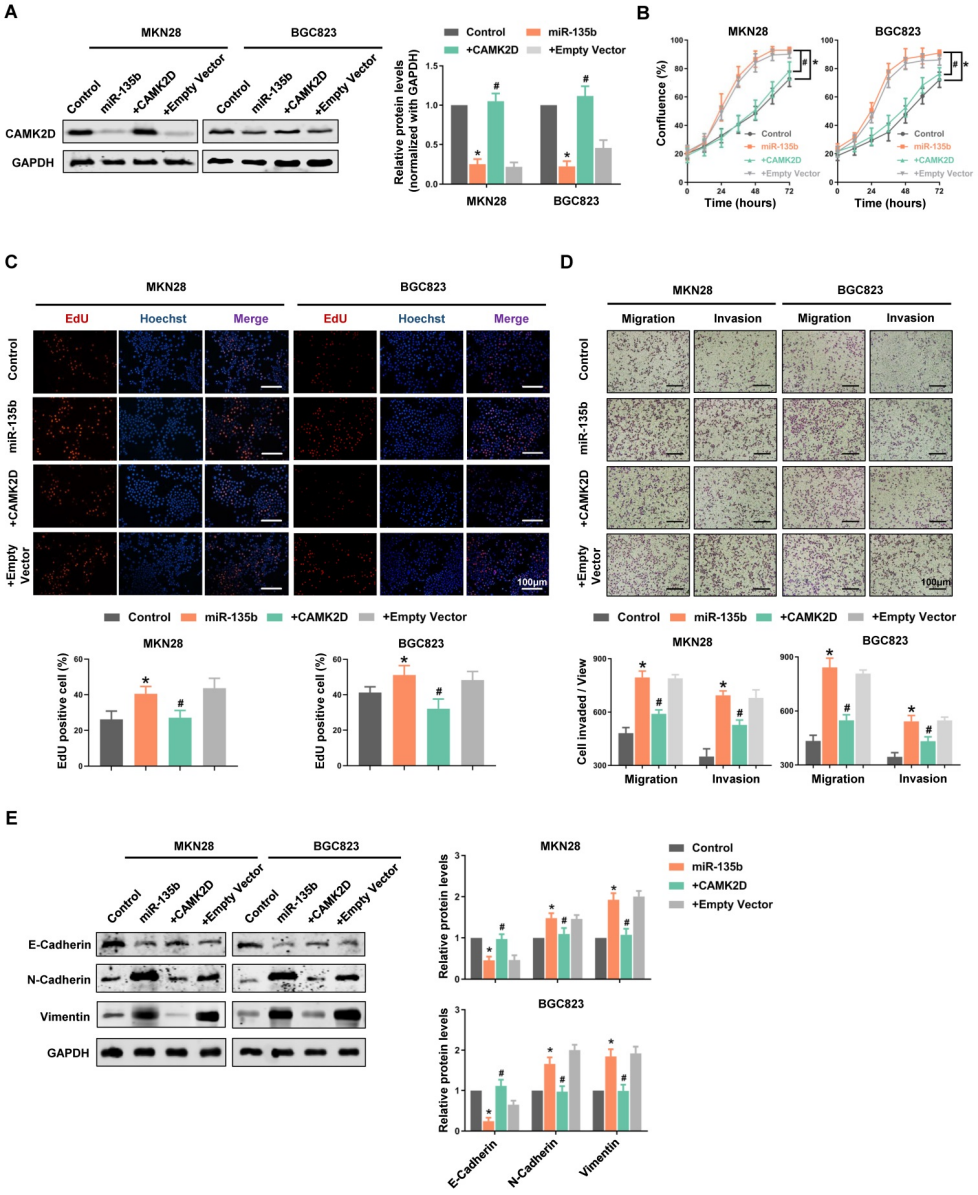
Restoring the expression of CAMK2D interfered with miR-135b-related functions and remodeled EMT process. GC cells were co-transfected with miR-135b mimics and CAMK2D expression plasmid lacking the miR-135b target region. (A) Western blot analysis of CAMK2D expression after 48 hours' co-transfection. (B) The cell proliferation was monitored by the IncuCyte system. (C) The activity of DNA replication was detected by EdU staining. (D) Overexpression of CAMK2D inhibited miR-135b-induced GC cells migration and invasion, as assessed using the transwell assay. Scale bar as indicated. (E) Western blot analysis of EMT-related proteins E-Cadherin, N-Cadherin and Vimentin after 48 hours' co-transfection. GAPDH was used as an internal control. n=6 independent experiments. *P<0.05 *vs* Control, #*P*<0.05 *vs* miR-135b.

To further confirm that CAMK2D downregulation was responsible for the cell malignant phenotype-related effects of miR-135b, we performed “rescue experiments”. In these experiments, CAMK2D protein was overexpressed from a plasmid lacking the miR-135b response element; it was therefore resistant to miRNA-mediated downregulation. Hence, although overexpression of miR-135b decreased endogenous CAMK2D protein levels significantly, both MKN28 and BGC823 cells co-transfected with the miRNA and the CAMK2D plasmid possessed near physiological levels of total CAMK2D protein (Figure 5A). Under these conditions, the enhancement of GC cells proliferation and an increase in the activity of DNA replication induced by miR-135b overexpression were blocked by upregulation of CAMK2D (Figure 5B and 5C). Moreover, the miR-135b-induced cell migration and invasion were also reversed upon co-expression of CAMK2D (Figure 5D). These results indicated that CAMK2D was the rate-limiting target of miR-135b to promote cell proliferation and migration.

Additionally, we detected the expression level of EMT-related proteins. The expression of epithelial cell marker E-cadherin was inhibited, whereas the expression of the mesenchymal cell marker N-cadherin and the cytoskeletal protein Vimentin were promoted in miR-135b overexpression cells. And, the miR-135b-mediated effects on the EMT-related proteins expression were reversed upon co-expression of CAMK2D (Figure 5E). The above results indicated that miR-135b targeted CAMK2D to regulate the EMT process, thereby promoted malignant progression of GC.

### Dysregulation of miR-135b affected the tumor progression of GC *in vivo*

**Figure 6:**
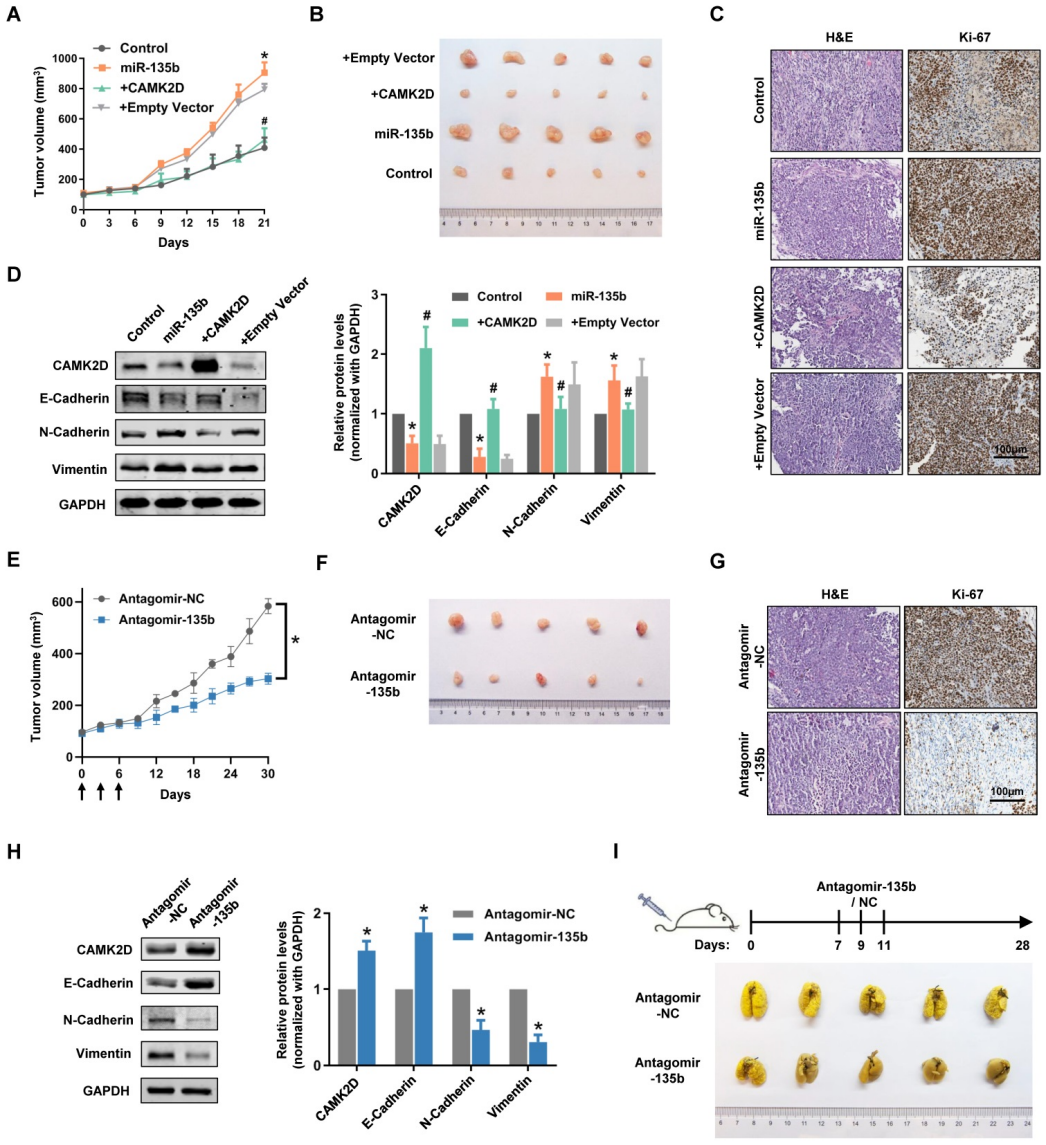
Dysregulation of miR-135b affected the tumor progression of GC *in vivo*. BGC823 cells were transfected with miR-135b mimics, miR-135b mimics+CAMK2D plasmid, miR-135b mimics+empty vector. And then, these cells were xenograft subcutaneously into the immune-deficient nude mice (n=5 for each group). (A) Tumor growth curves in xenograft formation assay. (B) The representative images of xenograft tumors dissected from the nude mice. (C) H&E staining (left panel) and IHC staining of Ki-67 (right panel). Scale bar as indicated. (D) Western blot analysis of CAMK2D and EMT-related proteins in the xenograft tumor tissues. (E) Antagomir-135b or antagomir-NC was delivered into mice body by tail vein injection once every three days from day 0 to 6 when the xenograft models established, and tumor growth curves were shown (n=5 for each group). The arrows indicate the days of injection. (F) The representative images of xenograft tumors. Antagomir-135b significantly inhibits the tumor growth of xenograft models compared with antagomir-NC. (G) H&E staining (left panel) and IHC staining of Ki-67 (right panel). Scale bar as indicated. (H) Western blot analysis of CAMK2D and EMT-related proteins in the xenograft tumor tissues from the mice injected with either antagomir-135b or antagomir-NC. GAPDH was used as an internal control. n=6 independent experiments. *P<0.05 *vs* Control or Antagomir-NC, #*P*<0.05 *vs* miR-135b. (I) Schematic representation of protocol used to study the ability of antagomir-135b to inhibit experimental tumor metastasis (top panel), and the representative images of lungs metastatic colonization by GC cells (bottom panel). Antagomir-135b significantly suppressed distant metastatic colonization.

To extend our observations from cell lines, we further investigated the role of miR-135b in GC xenografts models. As expected, miR-135b overexpression GC cells-injected nude mouse group showed faster tumor growth and bigger tumor volume compared with control nude mouse group (Figure 6A and 6B). More importantly, the tumors exhibited a slower growth rate and smaller size compared the co-expression of miR-135b and CAMK2D GC cells-injected group to the miR-135b overexpression GC cells-injected group. Immunohistochemical staining showed that the percentage of Ki-67-positive cells was markedly increased in the tumors from miR-135b overexpression group compared to controls, and was reversed upon introduction of CAMK2D overexpression plasmid (Figure 6C). We further detected the expression level of CAMK2D and EMT-related proteins in tumor tissues derived from xenografts models. The differential expression levels of CAMK2D and EMT-related proteins were similar to the results of *in vitro* experiments (Figure 6D). In other words, ectopic expression of the CAMK2D protein was sufficient to inhibit GC malignant progression even in the presence of miR-135b.

To explore whether miR-135b had a therapeutic potential in the treatment of GC, we performed tail vein injection of antagomir-135b once every three days from day 0 to 6 when the xenograft models established, and analyzed the tumor growth curves. Under this condition, antagomir-135b significantly inhibited the tumor growth of xenograft models (Figure 6E and 6F). More importantly, it was still able to attenuate tumor growth after two weeks of the injection of antagomir-135b, although the effects were less than earlier time points. Consistent with our previous results, the percentage of Ki-67-positive cells was decreased in the tumor tissue section of antagomir-135b group (Figure 6G). We also confirmed that inhibition of miR-135b* in vivo* induced an increase in the level of CAMK2D protein and modulated the expression of EMT-related proteins (Figure 6H). Metastatic potential was further assessed by counting colonized tumor nodules in the lung and only slight metastasis was found in mice injected with antagomir-135b (Figure 6I). These results suggested that antagomir-135b could be considered as a promising agent for the development of GC treatment.

## Discussion

Although much research has described the functions of miRNAs in GC, the molecular mechanism and clinical significance of miRNAs in GC have not been completely understood. In the present study, we identified a key regulator miRNA of GC, miR-135b through bioinformatics analysis. Our results indicated that miR-135b was upregulated in GC tissues compared to adjacent normal tissues. More importantly, the increase of miR-135b was also detected in the serum of GC patients, suggested that miR-135b could be used as a biomarker for early diagnosis of GC. Further *in vitro* and *in vivo* functional assays validated that miR-135b promoted GC progression and metastasis through directly targeting CAMK2D, a newly discovered tumor suppressor gene. Notably, we highlighted that the miR-135b antagomir could have therapeutic potential for GC treatment.

miR-135b is located on chromosome 1q32.1. Recent studies have implicated 1q32 genomic aberrations in lung cancer 27. Targeting the miR-135b cluster, which lead to its transcriptional activation, was likely due to amplifying its sequence. At present, upregulated miR-135b has been reported in a variety of cancers, and it has been shown to drive multiple malignant phenotypes 28-30. The amplification of miR-135b in colorectal cancer cells enhanced the chemotherapy resistance for doxorubicin, oxaliplatin, and 5-FU 31, 32. However, a few studies showed that overexpression of miR-135b suppressed tumor metastasis and increased sensitivity of chemotherapy in pancreatic cancer and breast cancer 33, 34. These could be partially explained by the mechanism that miRNA binding to mRNAs was often achieved with imperfect complementarity, and its targets varied in different cellular contexts and tumor types. We provided several lines of evidence that supported miR-135b function as an oncogene through targeting CAMK2D in GC. First, miR-135b was upregulated in GC cell lines, tissues as well as the serum samples of GC patients. Second, overexpression of miR-135b promoted GC cell proliferation, migration and invasion. In contrast, inhibition of miR-135b suppressed GC malignant progression both *in vitro* and *in vivo*. Third, the direct binding site between miR-135b and CAMK2D was confirmed by luciferase reporter analysis, and the miR-135b-induced tumor progression was partially antagonized by CAMK2D ectopic expression. In future works, we will concentrate on defining the reason that miR-135b is upregulated in GC and the translational application of miR-135b in clinical diagnosis and treatment.

At present, we demonstrated CAMK2D was a functional downstream target of miR-135b. CAMK2D is a novel tumor suppressor, which has been found to be inactivated in several types of cancers, including colorectal cancer, prostate cancer, and lung cancer 35-37. CAMK2D was frequently decreased in colorectal cancer and was associated with poor prognosis 38. High expression of CAMK2D is conducive to restrain cell proliferation and decrease cell survival in GC. Likewise, the online data suggested that low level of CAMK2D was also associated with poor prognosis of GC patients. However, the mechanism underlying the loss of CAMK2D has not been illustrated. Based on bioinformatics prediction, we found that CAMK2D may be a potential target of miR-135b, which bound to the 3'-untranslated region (UTR) of CAMK2D mRNA and inhibited the expression of CAMK2D at both the transcriptional and post-transcriptional levels. In this study, we showed that the expression levels between CAMK2D and miR-135b were significantly negatively correlated in GC. And we further confirmed CAMK2D was the direct target of miR-135b. Although our results demonstrated that the lack of CAMK2D in GC was mainly inhibited by miR-135b overexpression, we did not exclude the possibility that CAMK2D could also be modulated by other mechanisms, such as loss-of-function mutation, copy number depletion or hyper-methylation.

Previous studies have shown that overexpression of CAMK2D inhibits proliferation, migration and invasion in cancer cells. CAMK2D has also been found to be a downstream effector of interferon-γ (IFN-γ), indicating that CAMK2D may be involved in the regulation of tumor immune microenvironment 39. Interestingly, CAMK2D can be targeted by miR-146a and is consistently shown to suppress the expression of matrix-related genes, indicated that CAMK2D may also be involved in the EMT process in tumor cells 40. Our study showed that CAMK2D acted as a tumor suppressor that interfered with miR-135b-induced GC cells migration and invasion. Importantly, we first discovered that CAMK2D participated in the regulation of EMT process, since overexpression of CAMK2D affected EMT-related proteins expression. However, due to the limitation of current study, we did not identify the specific relationship between CAMK2D and the EMT process.

In conclusion, we revealed the upregulation of miR-135b in GC, proved it a potential biomarker for GC through integrated analyses. Functional assays demonstrated that miR-135b promoted GC progression both *in vitro* and *in vivo*. Additionally, we identified CAMK2D as a direct target of miR-135b, and was essential for mediating the oncogenic effects of miR-135b in GC cells. At last, we found that inhibition of endogenous miR-135b by the antagomir suppressed tumor progression in xenograft models. Therefore, this research demonstrated that targeting miR-135b/CAMK2D axis could be a potential novel strategy for GC treatment.

## Materials and Methods

### Clinical specimen collection

A total of de-identified 146 GC tissue samples and 28 pairs of GC tissue samples and adjacent normal tissue samples were obtained from the Biobank of Peking University Cancer Hospital. None of the patients received chemotherapy or radiation therapy prior to surgery. The histology and age information are included in Supplementary Table S1.

Blood samples from 23 GC patients and 27 health controls were collected according to standard phlebotomy procedures. The histology and age information are included in Supplementary Table S2. A total of 10 mL of whole blood from each participant was collected into tubes and immediately placed on ice. Blood samples were subjected to centrifugation at 1000×g for 10 minutes at 4°C to spin down the blood cells. The serum supernatant was removed by pipette from the cellular material and then aliquoted and stored in liquid nitrogen tanks until assays were performed.

Written informed consent was provided by all patients, in compliance with Declaration of Helsinki. The Ethics Committee of the Peking University Beijing Cancer Hospital approved all aspects of this research (Permission number 2019KT111).

### Cell culture

The GC-derived cell lines MKN28 and BGC823, and the normal gastric mucosa-derived cell line GES-1 were obtained from Chinese National Infrastructure of Cell Line Resource. MKN28 and BGC823 cells were seeded in Roswell Park Memorial Institute 1640 medium (RPMI 1640; GIBCO, USA), and GES-1 cells was seeded in Dulbecco's Modified Eagle medium (DMEM; GIBCO, USA). All mediums were supplemented with 10% fetal bovine serum (FBS; GIBCO, USA). All cells were cultured at 37°C with 5% CO_2_ in a humidified incubator.

### Cell transfection

The miR-135b mimics, inhibitor, and negative control (NC) were obtained from RiboBio (China). CAMK2D overexpression plasmid and empty vector were generated by Genechem (China). We seeded cells into a 6-well plate, and the transfection with mimics, inhibitor, and expression plasmid was performed after 24 hours using Lipofectamine 2000 (Invitrogen, USA), according to the manufacturer's protocols. In each well, a final concentration of 100 nM miRNA mimics, inhibitor, or 1 μg plasmid were added as described. The transfection efficiency was determined by using qRT-PCR.

### RNA extraction and qRT-PCR

Total RNA was extracted from tissue samples and cell lines using TRIzol Reagent (Invitrogen, USA). according to the manufacturer's instructions. First-strand cDNA was generated by RT-PCR using a reverse transcription system kit (Invitrogen, USA). qRT-PCR was performed with the ABI PRISM 7500 Sequence Detection System according to the SYBR Green method. All reactions were performed at least three times. Cycle threshold (CT) values were determined using fixed threshold settings after completion of the reaction. U6 small nuclear RNA was used as an internal control for miR-135b, and the CAMK2D mRNA levels were normalized to those of GAPDH. Their relative expression levels normalized to the control were further calculated using the 2^ΔΔ-ct^ method. The primers used are listed in Supplementary Table S3.

### Western blot analysis

We obtained protein samples from GC tissues and cells using RIPA lysis buffer. The samples were separated through a 12.5% SDS-PAGE and the NC membranes were blocked with 5% skim milk in at room temperature for 1 hour. Next, we incubated the membranes overnight at 4°C with EMT markers (E-cadherin, N-cadherin, Vimentin), CAMK2D, and GAPDH antibodies. The image of protein bands and the quantification of expression levels were measured by Odyssey system (LI-COR Biosciences, USA). The information for primary antibodies was as follows, CAMK2D (1:500, #ab181052, Abcam, USA), GAPDH (1:1000, #60004-1-Ig, Proteintech, China), EMT antibody sampler kit (1:1000, #9782, Cell signaling technology, USA). The secondary antibodies, IRDye 800CW goat anti-mouse (1:5000, #926-32210, LI-COR Biosciences, USA) and donkey anti-rabbit (1:5000, #926-32213, LI-COR Biosciences, USA).

### Colony formation assay

For the colony formation assay, MKN28 and BGC823 cells were cultured into 6-well plates at 600 cells/well and incubated at 37°C with 5% CO_2_ for two weeks. Then, GC cells were washed three times with PBS and fixed with methanol for 15 minutes at room temperature. The cells were stained with 0.1% crystal violet, and the colonies containing >100 cells were count.

### EdU staining and cell proliferation assay

MKN28 and BGC823 cells were treated as indicated, washed three times with PBS, and then incubated in serum-free RPMI 1640 containing 10 μmol/L EdU (RiboBio, China) for 2 hours. Cells were fixed, then underwent Apollo staining and nuclear staining, according to the manufacturer's instructions. Finally, the cells were imaged by confocal microscope and the percentage of proliferating cells was further calculated.

To further determine the level of cell proliferation, the cells were seeded in 96-well plates at a density of 3×10^3^/well, and then monitored with IncuCyte live cell analysis imaging system (Essen Biosciences, USA).

### Cell migration and invasion assay

We tested the capacity of GC cells migration and invasion using a transwell chambers (8 μm pore size membranes). The lower chamber was added with 10% FBS and incubated at 37°C with 5% CO_2_. Then the upper surface with matrigel (BD Biosciences, USA) was used for cell invasion. And cell migration assay was conducted without matrigel. After the transfection of GC cells for 36 hours, these cells were seeded in the upper chamber with serum-free medium. 48 hours later, the migrated or invasive cells were fixed with methanol and stained with 0.1% crystal violet. We obtained images of migrated cells by using a microscope, and the cell numbers of migration and invasion were counted by using Image Pro Plus.

### Dual luciferase assay

Wild-type and mutant of CAMK2D 3'-UTR were synthesized and inserted into the p-MIR-REPORT plasmid (GenePharma, China) to perform luciferase reporter experiments. Then, wild or mutant type of 3'-UTR of CAMK2D and miR-135b mimics were transfected into HEK293T cells. Subsequently, the luciferase activity was measured through luciferase assay kit (Promega, USA).

### Statistical analysis

All data were representative of at least three independent experiments. The data were presented as mean values ± standard deviation (SD). The difference between the groups was calculated through Tukey's one-way ANOVA or two-tailed Student's *t*-test. The survival curves were drawn by Kaplan-Meier analysis, and log-rank test was used to compare the survival differences. Differences with a value of *P*<0.05 was regarded as statistically significant. Statistical analyses were performed by GraphPad Prism 6.0 software (GraphPad Software Inc., USA).

## Supplementary Material

Supplementary figures and tables.
