# Supplementary material for: MicroRNA-135b/CAMK2D Axis Contribute to Malignant Progression of Gastric Cancer through EMT Process Remodeling
Source: Int J Biol Sci. 2021 May 10;17(8):1940–52. doi: 10.7150/ijbs.58062 (PMC8193265; doi:10.7150/ijbs.58062)
Supplement: Supplementary file 1 — Supplementary figures and tables. [file ijbsv17p1940s1.pdf]

Supplementary Methods

Public data query and mining

Gene and microRNA expression profiles by RNA-seq for cohorts of 428 cases stomach adenocarcinoma (STAD), 527 cases brain lower grade glioma (LGG), 496 cases kidney renal clear cell carcinoma (KIRC), 177 cases pancreatic adenocarcinoma (PAAD) that have clinical outcome data available were extracted from The Cancer Genome Atlas (TCGA). All expression values were converted into $\log_2$(TPM+1). MicroRNA expression datasets GSE23739, GSE26595, GSE28700, GSE93415 and gene expression dataset of Asian Cancer Research Group (ACRG; GSE66229) were extracted from the GEO database. We analyzed the relationship between microRNAs and the prognosis of cancer patients using Kaplan–Meier method. The cutoff point of each microRNA expression was determined by X-Tile software (Rimm Lab, USA).

Bioinformatics analysis

The target genes of miR-135b were predicted by TargetScan V7.2 (http://www.targetscan.org). And, we further performed gene ontology (GO) enrichment analysis, which was a useful method for annotating the target genes of microRNA with biological characteristics. miR-135b and its targets interaction network were visualized by Cytoscape 3.7.2 (https://www.cytoscape.org/), which could provide information on the molecular mechanism underlying biological process. The gene expression levels of the network were analyzed using Peking University Cancer Hospital Gastric Cancer Transcriptome Dataset (PUCH dataset; DOI: 10.21147/j.issn.1000-9604.2019.05.07). All data analyses were performed in the R programming and JAVA environment.

Immunohistochemistry (IHC) staining

Tissues from GC patients and xenograft tumors were fixed in 4% paraformaldehyde and embedded in paraffin blocks. The tissues were embedded into paraffin as blocks for storage and 5-μm-thick sections were cut into glass slides. Sections were incubated with primary antibody at 4°C overnight and then incubated with a secondary antibody at room temperature for 1 hour. After washing the slides, chromogen was applied and images were obtained with the digital pathology scaner (Aperio CS2, Leica Biosystems, USA). The primary Ki-67 antibody (#ZM-0166, ZSGB-BIO, China) and CAMK2D antibody (1:200, #15443-1-AP, Proteintech, China) were used.

Functional assay *in vivo*

Female BALB/c nude mice (Five-weeks old) were purchased from Beijing Vital River Laboratory Animal Technology Co. Ltd. Subcutaneous xenograft tumor model was used to estimate the effects of miR-135b on tumorigenicity *in vivo*. 2×10^6 BGC823 cells were transfected with miR-135b mimics or cotransfected with miRNA mimics and either CAMK2D overexpression plasmid or empty vector. After 24 hours, these cells were resuspended in PBS with 50% Matrigel and engrafted subcutaneously into each flank of nude mice. Antagomir-135b and antagomir-NC were obtained from RiboBio (China), tail vein injection three times, 100nM per mouse. The tumors observed in mice were measured every 3 days for three weeks. The tumor volume was calculated according to the formula: $length\times width^2/2$. At the end of experiment, the mice were sacrificed and the

tumors were collected. The dissected tissues were formalin-fixed and paffin-embedded for hematoxylin-eosin (H&E) staining, and Ki-67 IHC staining.

For experimental tumor metastatic model, BGC823 cells (5×10^6 cells in a 100 μL volume per mouse) were injected into the tail vein of BALB/c nude mice. After 1 week, these mice were randomly divided into two groups: antagomir-135b and antagomir-NC. Mice were treated either antagomir-135b or antagomir-NC (tail vein injection 3 times, 100nM per mouse). Four weeks later, the mice were sacrificed and the lungs were excised from the body. Bouin's solution was injected from the main bronchi to fix the lung tissues. All animal experiments were conducted in accordance with the Institutional Animal Care and Use Committee guidelines at Peking University Beijing Cancer Hospital.

Supplementary Data

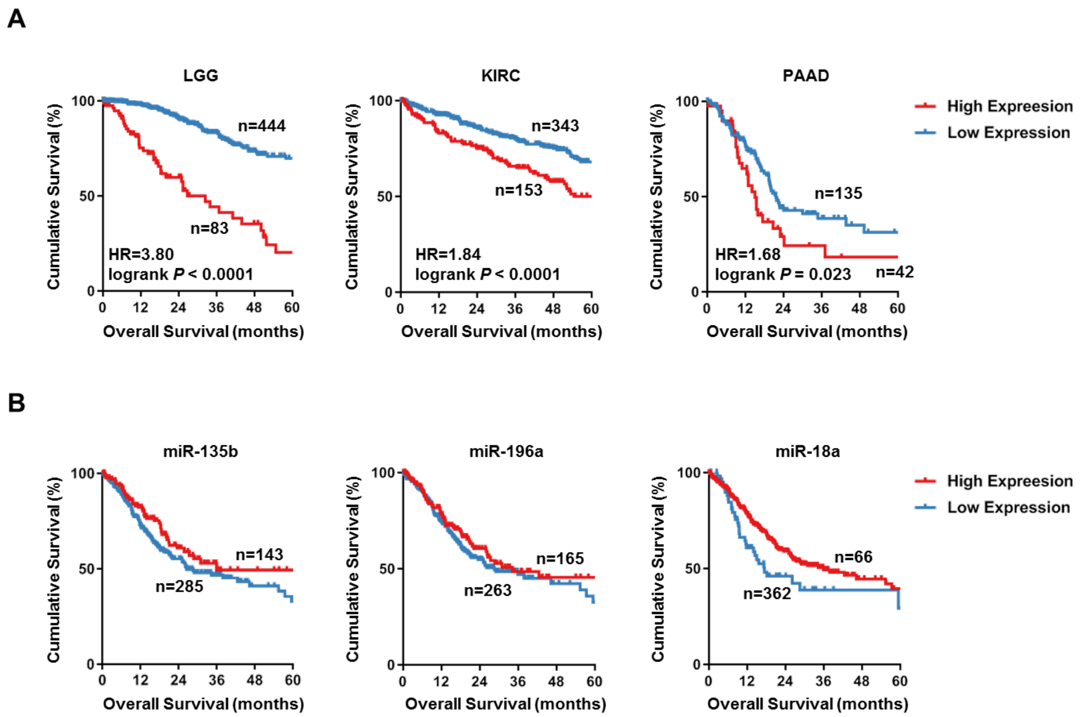

Figure S1. Survival analysis using TCGA data. (A) The high expression level of miR-135b was significantly associated with poor prognosis in patients with brain lower grade glioma (LGG), kidney renal clear cell carcinoma (KIRC), pancreatic adenocarcinoma (PAAD). (B) No prognostic significance in patients with gastric cancer was found among miR-135b, miR-196a and miR-18a. HR: hazard ratio.

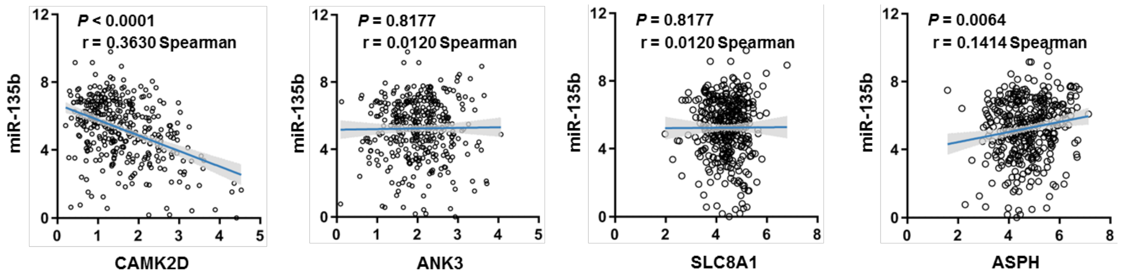

Figure S2. Correlation analysis of miR-135 and its predicted targets using TCGA data. The results showed that the expression levels between miR-135b and CAMK2D were significantly negatively correlated.

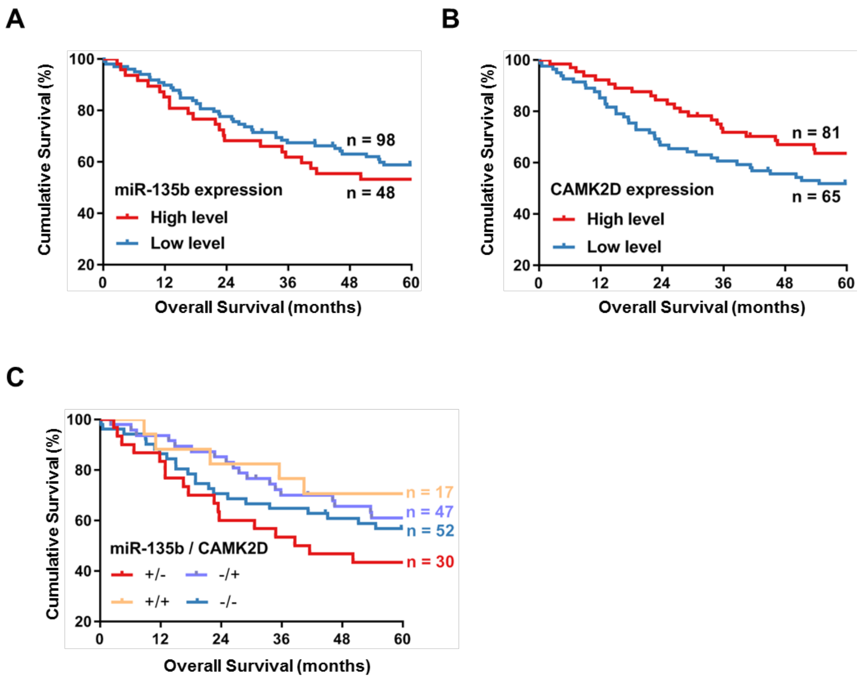

Figure S3. Survival analysis in GC patients based on expression levels of miR-135b and its target CAMK2D.

Supplementary Tables

Table S1. Pathological information of human gastric tissue samples for miRNA and mRNA qRT-PCR validation.

Sample ID	Gender	Age	TNM	Stage	Lauren classification	Histological grade
1	M	66	T3N3M0	IIIB	Diffuse	Poor
2	M	51	T3N3M0	IIIB	Intestinal	Moderate
3	F	47	T3N2M0	IIIA	Missing	Moderate
4	M	59	T3N3M0	IIIB	Intestinal	Moderate
5	M	53	T2N2M0	IIB	Mixed	Poor
6	M	55	T3N3M0	IIIB	Diffuse	Missing
7	M	56	T3N2M0	IIIA	Intestinal	Moderate
8	F	74	T3N0M0	IIA	Diffuse	Poor
9	M	58	T3N0M0	IIA	Intestinal	Moderate
10	M	55	T4N1M0	III	Diffuse	Poor
11	M	58	T3N2M0	IIIA	Intestinal	Moderate
12	M	78	T2N1M0	IIA	Mixed	Moderate
13	F	50	T2N3M0	IIIA	Intestinal	Moderate
14	M	42	T3N1M0	IIB	Diffuse	Poor
15	F	32	T3N3M0	IIIB	Diffuse	Poor
16	M	68	T3N3M0	IIIB	Mixed	Moderate
17	M	66	T3N1M0	IIB	Mixed	Poor
18	M	69	T3N3M0	IIIB	Diffuse	Poor
19	F	60	T3N2M1	IV	Intestinal	Poor
20	M	59	T2N2M0	IIB	Mixed	Poor
21	M	55	T3N1M0	IIB	Diffuse	Poor
22	F	51	T4N3M0	III	Diffuse	Poor
23	M	43	T3N1M0	IIB	Mixed	Moderate
24	F	41	T3N2M0	IIIA	Intestinal	Moderate
25	F	45	T3N3M0	IIIB	Diffuse	Poor
26	M	49	T2N0M0	IIB	Mixed	Moderate
27	M	75	T3N0M0	IIA	Intestinal	Moderate
28	M	69	T3N3M0	IIIB	Diffuse	Poor
29	M	75	T3N1M0	IIB	Mixed	Moderate
30	M	75	T3N0M0	IIA	Intestinal	Moderate
31	M	77	T3N3M0	IIIB	Intestinal	Moderate
32	M	45	T3N0M0	IIA	Diffuse	Poor
33	M	64	T3N3M0	IIIB	Diffuse	Missing
34	M	55	T1N0M0	IA	Intestinal	Moderate
35	M	44	T3N1M0	IIB	Intestinal	Moderate

36	M	60	T3N1M0	IIB	Intestinal	Moderate
37	M	73	T3N3M0	IIIB	Mixed	Moderate
38	F	53	T2N0M0	IB	Mixed	Moderate
39	F	57	T3N0M0	IIA	Diffuse	Poor
40	M	56	T3N2M0	IIIA	Diffuse	Poor
41	M	38	T3N3M0	IIIB	Diffuse	Moderate
42	F	56	T3N0M0	IIA	Diffuse	Poor
43	M	62	T2N0M0	IB	Intestinal	Well
44	F	48	T2N0M0	IB	Mixed	Moderate
45	F	70	T2N1M0	IIA	Mixed	Poor
46	M	54	T3N0M0	IIA	Intestinal	Moderate
47	M	80	T3N2M0	IIIA	Diffuse	Poor
48	M	65	T3N1M0	IIB	Mixed	Moderate
49	M	39	T3N0M0	IIA	Diffuse	Missing
50	M	59	T3N3M0	IIIB	Diffuse	Poor
51	F	53	T3N3M0	IIIB	Mixed	Poor
52	F	74	T3N0M0	IIA	Intestinal	Poor
53	M	61	T2N2M0	IIB	Intestinal	Poor
54	M	72	T3N1M0	IIB	Intestinal	Moderate
55	M	49	T3N1M0	IIB	Intestinal	Moderate
56	F	64	T4N3M0	III	Diffuse	Poor
57	F	58	T1N0M0	IA	Diffuse	Poor
58	M	71	T2N1M0	IIA	Intestinal	Poor
59	M	70	T2N1M0	IIA	Intestinal	Missing
60	M	60	T2N1M0	IIA	Intestinal	Moderate
61	M	63	T1N0M0	IA	Mixed	Poor
62	M	67	T4N2M0	III	Intestinal	Poor
63	M	54	T4N0M0	IIB	Missing	Poor
64	M	76	T3N3M0	IIIB	Intestinal	Moderate
65	M	66	T4N3M0	III	Missing	Poor
66	M	56	T3N0M0	IIA	Mixed	Moderate
67	F	55	T3N0M0	IIA	Diffuse	Poor
68	M	67	T3N3M0	IIIB	Mixed	Moderate
69	M	52	T3N3M0	IIIB	Intestinal	Moderate
70	M	41	T3N0M0	IIA	Mixed	Moderate
71	M	36	T3N0M0	IIA	Diffuse	Poor
72	M	60	T4N2M0	III	Diffuse	Poor
73	M	51	T3N3M0	IIIB	Intestinal	Poor
74	F	69	T3N1M0	IIB	Mixed	Moderate
75	M	59	T3N3M0	IIIB	Intestinal	Well
76	M	48	T3N3M0	IIIB	Diffuse	Poor

77	M	46	T4N1M0	III	Mixed	Poor
78	F	41	T3N2M0	IIIA	Mixed	Moderate
79	M	79	T3N3M0	IIIB	Intestinal	Moderate
80	M	54	T3N1M0	IIB	Intestinal	Well
81	M	52	T3N1M0	IIB	Intestinal	Moderate
82	F	52	T4N1M0	III	Intestinal	Moderate
83	F	64	T3N3M0	IIIB	Mixed	Moderate
84	M	66	T2N0M0	IB	Intestinal	Moderate
85	M	79	T3N2M0	IIB	Diffuse	Poor
86	M	57	T3N2M0	IIB	Mixed	Poor
87	M	56	T2N0M0	IB	Intestinal	Moderate
88	F	57	T3N0M0	IIA	Intestinal	Moderate
89	M	41	T3N2M0	IIIA	Diffuse	Poor
90	F	56	T1bN0M0	IA	Mixed	Poor
91	M	80	T3N3M0	IIIB	Intestinal	Moderate
92	M	69	T2N3M0	IIIA	Intestinal	Moderate
93	M	61	T3N0M0	IIA	Intestinal	Missing
94	M	59	T3N3M0	IIIB	Intestinal	Poor
95	M	45	T1bN0M0	IA	Diffuse	Poor
96	M	58	T2N3M0	IIIA	Diffuse	Poor
97	M	55	T3N2M0	IIIA	Diffuse	Poor
98	M	62	T3N2M0	IIIA	Intestinal	Moderate
99	M	51	T2N0M0	IB	Intestinal	Well
100	F	68	T4N2M0	III	Intestinal	Moderate
101	M	42	T3N2M0	IIIA	Intestinal	Moderate
102	F	53	T3N2M0	IIIA	Diffuse	Poor
103	F	34	T2N0M0	IB	Missing	Moderate
104	M	49	T3N3M0	IIIB	Missing	Moderate
105	M	71	T3N0M0	IIA	Missing	Moderate
106	M	75	T3N2M0	IIIA	Missing	Moderate
107	M	61	T2N2M0	IIB	Missing	Moderate
108	F	58	T3N2M0	IIIA	Missing	Moderate
109	F	67	T2N2M0	IIB	Intestinal	Moderate
110	M	59	T3N0M0	IIA	Intestinal	Moderate
111	F	52	T3N1M0	IIB	Intestinal	Moderate
112	M	82	T2N3M0	IIIA	Diffuse	Poor
113	M	74	T3N1M0	IIB	Intestinal	Moderate
114	F	52	T3N2M0	IIIA	Intestinal	Moderate
115	M	57	T3N1M0	IIB	Mixed	Poor
116	M	62	T3N3M0	IIIB	Mixed	Poor
117	M	69	T3N0M0	IIA	Intestinal	Moderate

118	M	62	T3N3M0	IIIB	Diffuse	Poor
119	F	79	T2N0M0	IB	Intestinal	Poor
120	M	58	T3N3M0	IIIB	Mixed	Poor
121	M	36	T2N2M0	IIB	Diffuse	Poor
122	M	46	T3N3M0	IIIB	Diffuse	Poor
123	M	52	T3N0M0	IIA	Intestinal	Moderate
124	M	60	T4N2M0	IIIB	Missing	Moderate
125	F	71	T3N2M0	IIIA	Mixed	Moderate
126	F	81	T4N3M1	IV	Mixed	Moderate
127	F	46	T3N2M0	IIIA	Diffuse	Poor
128	M	74	T2N1M0	IIA	Intestinal	Moderate
129	F	62	T2N3M0	IIIA	Diffuse	Poor
130	M	47	T2N0M0	IB	Diffuse	Poor
131	F	71	T2N0M0	IB	Mixed	Poor
132	M	69	T3N0M0	IIA	Diffuse	Poor
133	M	57	T3N2M0	IIIA	Intestinal	Moderate
134	M	63	T3N3M0	IIIB	Intestinal	Moderate
135	M	57	T3N3M0	IIIB	Mixed	Moderate
136	M	50	T3N1M0	IIB	Mixed	Moderate
137	M	64	T4N2M0	III	Intestinal	Moderate
138	M	71	T2N0M0	IB	Mixed	Poor
139	M	33	T3N2M0	IIIA	Mixed	Poor
140	M	69	T3N0M0	IIA	Intestinal	Moderate
141	M	56	T3N3M0	IIIB	Intestinal	Moderate
142	M	42	T3N0M0	IIA	Mixed	Moderate
143	M	52	T3N3M0	IIIB	Mixed	Moderate
144	F	64	T3N3M0	IIIB	Intestinal	Moderate
145	F	60	T3N1M0	IIB	Missing	Moderate
146	M	56	T3N3M0	IIIB	Missing	Moderate

Table S2. Pathological information of blood samples for miRNA qRT-PCR validation.

Sample ID	Gender	Age	Pathology/diagnosis
1	M	68	Adenocarcinoma
2	M	52	Adenocarcinoma
3	F	33	Adenocarcinoma
4	M	71	Adenocarcinoma
5	M	54	Adenocarcinoma
6	M	68	Adenocarcinoma
7	M	57	Adenocarcinoma
8	M	64	Adenocarcinoma
9	M	57	Adenocarcinoma
10	M	64	Adenocarcinoma
11	F	57	Adenocarcinoma
12	M	65	Adenocarcinoma
13	F	67	Adenocarcinoma
14	M	53	Adenocarcinoma
15	M	69	Adenocarcinoma
16	M	50	Hepatic metastases from GC
17	F	74	Adenocarcinoma
18	M	71	Adenocarcinoma
19	M	72	Adenocarcinoma
20	M	69	Suspected hepatoid adenocarcinoma
21	M	48	Adenocarcinoma
22	F	45	Adenocarcinoma
23	M	58	Adenocarcinoma
24	M	37	Normal
25	M	44	Normal
26	M	56	Normal
27	M	61	Normal
28	F	65	Normal
29	F	60	Normal
30	F	55	Normal
31	M	47	Normal
32	M	48	Normal
33	M	67	Normal
34	M	68	Normal
35	F	70	Normal
36	M	66	Normal
37	M	54	Normal

38	M	55	Normal
39	M	49	Normal
40	M	60	Normal
41	F	41	Normal
42	F	43	Normal
43	F	51	Normal
44	F	47	Normal
45	M	62	Normal
46	F	54	Normal
47	M	53	Normal
48	F	49	Normal
49	M	53	Normal
50	M	51	Normal

Table S3. Primers list for miRNA and mRNA qRT-PCR validation.

Primer		Sequence (5’--------------3’)
miR-135b-5p	Forward	ACACTCCAGCTGGGTATGGCTTTCATTCCT
	Reverse	TGGTGTCGTGGAGTCG
miR-196a-5p	Forward	ACACTCCAGCTGGGTAGGTAGTTTCATGTT
	Reverse	TGGTGTCGTGGAGTCG
miR-18a-5p	Forward	ACACTCCAGCTGGGTAAGGTGCATCTAGTGC
	Reverse	TGGTGTCGTGGAGTCG
Let-7e	Forward	ACACTCCAGCTGGGTGAGGTAGTAGGTTGT
	Reverse	TGGTGTCGTGGAGTCG
U6	Forward	CTCGCTTCGGCAGCACATATACT
	Reverse	ACGCTTCACGAATTTGCGTGTC
CAMK2D	Forward	AGGGCTTTCACTACTTGGTGT
	Reverse	AGCCAAAGTCTGCCAATTTCA
GAPDH	Forward	GACTCATGACCACAGTCCATGC
	Reverse	AGAGGCAGGGATGAT GTTCTG
